# Perspectives of epidemiological control of the MRSA subpopulations in hospital patients with sepsis via molecular detection, genetic analysis and following typing of methicilin resistance-carrying mobile genetic elements SCC*mec* in Ukraine

**DOI:** 10.1186/1878-5085-5-S1-A132

**Published:** 2014-02-11

**Authors:** Olena V Moshynets, Svitlana U Rymar, Olga B Liutko, Nadiia M Oserjanskaja, Gennadii B Kolov, Nadiia P Kisyl, Rostyslav V Bubnov, Maria Boretska, Dmytro M Irodov

**Affiliations:** 1 Institute of Molecular Biology and Genetics of NAS of Ukraine, Kyiv, Ukraine; 2 Institute of Traumatology and Orthopaedics of the NAMS of Ukraine, Kyiv, Ukraine; 3 National Children Specialized Hospital “OCHMATDYT”, Kyiv, Ukraine; 4 Zabolotny Institute of Microbiology and Virology of NAS of Ukraine, Kyiv, Ukraine

## Background

Sepsis and its after-effects are currently significant problems in hospital medicine. Successful treatment depends on the early detection of the etiological agent of the sepsis and of any antibiotic resistances. Bacteria that belong to *Staphylococcus* spp. are the most important Gram-positive causative agents of sepsis and are responsible for ~ 50% of sepsis and pyoinflammatory diseases in Ukraine. Up to 70% of Ukrainian staphylococci are also methicillin resistant which is determined by the genetic cassette SCC*mec* encoding the *mecA* β-lactam resistance gene. Besides *mecA*, some types of SCC*mec*s can also carry different genes of resistances to antibiotics like vancomycin etc. Until now nine types of SCC*mec* have been discovered based on the genetic construction, described and epidemiologically traced worldwide. It is now clear that new SCC*mec*s with enhanced antibiotic resistance characteristics are naturally appearing due to anthropogenic activities. Ukraine being geographically on a crossroads between Asia and Europe, having a big borderline with four countries-member of the EU and three non-EU European countries and exploiting a medical system where any antibiotic is easy to get without a doctor prescription can have a really dramatic impact on the SCC*mec* evolution in Europe. For this reason, a rapid screening assay with the following analysis of the SCC*mec*s is very much needed for the early identification of new SCC*mec*s in Ukraine and prediction of new epidemiological waves in Europe.

## The aim

was to elaborate a PCR rapid diagnostic test to reveal SCC*mec* in Ukrainian staphylococci.

## Materials and methods

Four PCR-based assays [1-4] for methicillin-resistance identification were tested using forty *Staphylococcus* spp. isolates obtained from patients of the Institute of Traumatology and Orthopaedics of the NAMS of Ukraine.

## Results

A new “in house” multiplex PCR test based on [3] for the direct simultaneous identification of SCC*mec* was developed and implemented.

## Conclusions

A rapid PCR-based test for β-lactam resistance identification is now available in Ukraine. A collection of Ukrainian MRSA from different regions with PCR-determined presence of SCC*mec* has been now created and constantly supplemented.

## Outlook and expert recommendations

We recommend to organize project based on direct bacteria identification using SCCmec for implementation rapid screening in Ukraine. The epidemiological monitoring of Ukrainian SCC*mec*s with the detailed analysis of new SCC*mec* variants may impact prediction of new epidemiological waves in Europe.
